# Depemokimab and the twice-yearly regimen: pharmacological implications and therapeutic positioning in severe eosinophilic respiratory diseases

**DOI:** 10.3389/fimmu.2026.1783889

**Published:** 2026-03-18

**Authors:** A. M. Marra, E. Bizzi, A. Gidaro, F. Bini, S. Rotunno, S. Modica, E. Greco, A. Mauro, R. Mascolo, E. Tombetti, M. Lazzeroni, F. Paciullo, G. Abatianni, C. Gagliardi, S. Damanti, P. Rovere Querini

**Affiliations:** 1 Pulmonology Unit, Azienda Socio-Sanitaria Territoriale (ASST) Rhodense, Garbagnate Milanese Hospital, Milan, Italy; 2 Department of Internal Medicine, Vita-Salute San Raffaele Hospital, Milan, Italy; 3 Department of Internal Medicine, Sacco Hospital, Azienda Socio-Sanitaria Territoriale (ASST) Fatebenefratelli and Sacco Hospitals, Milan, Italy; 4 Internal Medicine, S.Pietro Fatebenefratelli Hospital, Rome, Italy; 5 Rheumatology, Allergology and Clinical Immunology, Department of “Medicina dei Sistemi”, University of Rome Tor Vergata, Rome, Italy; 6 Department of Pediatrics, Fatebenefratelli Hospital, Milan, Italy; 7 Department of Internal Medicine, Fatebenefratelli Hospital, Azienda Socio-Sanitaria Territoriale (ASST) Fatebenefratelli and Sacco Hospitals, Milan, Italy; 8 Department of Otorhinolaryngology & Head and Neck Surgery, Fatebenefratelli Hospital, ASST Fatebenefratelli Sacco, Milan, Italy

**Keywords:** asthma, CRSwNP, depemokimab, IL-5, IL-5 blockage, positioing

## Abstract

Type 2 inflammatory diseases (T2D), notably severe eosinophilic asthma (SEA) and chronic rhinosinusitis with nasal polyps (CRSwNP), represent a significant global health burden due to their high morbidity, frequent exacerbations and reliance on systemic glucocorticoids (SGCs). Targeting Interleukin-5 (IL-5), a key driver of eosinophil production and survival, has emerged as a validated therapeutic strategy. Depemokimab is a novel, first-in-class anti-IL-5 monoclonal antibody (mAb) engineered with an extended half-life via the YTE amino acid modification of its Fc region, enabling an unprecedented twice-yearly dosing interval. This review synthesizes clinical data from the Phase III SWIFT (SEA) and ANCHOR (CRSwNP) programs, demonstrating depemokimab’s sustained eosinophil depletion and significant reduction in asthma exacerbation rates (annualized asthma exacerbation rate reduction of approximately 54% versus placebo). While efficacy on secondary endpoints such as lung function (FEV1) and quality of life (QoL) was mixed, the efficacy in reducing nasal polyp size (Nasal Polyp Score and Nasal Congestion) in CRSwNP was clear. The core value proposition of depemokimab is the combination of established IL-5 inhibition efficacy with patient convenience due to its dosing. We discuss its pharmacodynamic profile, safety and pivotal role in shifting the treatment paradigm towards simplified chronic disease management, positioning depemokimab as a novel, patient-centric option in the competitive landscape of T2 biologic therapies.

## 1 Introduction

### 1.1 The interconnected burden of type 2 inflammation

Type 2 inflammatory diseases (T2D) are driven by the activation of T-helper 2 (Th2) cells and the subsequent release of specific cytokines, primarily Interleukin-4 (IL-4), Interleukin-5 (IL-5), and Interleukin-13 (IL-13) (1). These complex and often overlapping pathologies, including severe eosinophilic asthma (SEA) and chronic rhinosinusitis with nasal polyps (CRSwNP), often coexist, creating a synergistic effect that amplifies the clinical and socioeconomic burden and complicating management strategies (2). The presence of type 2 (T2) comorbidities complicates disease management, reduces patient quality of life and drastically increases healthcare utilization costs, particularly hospitalization and emergency room visits (3). Addressing these diseases simultaneously is crucial for improving patient outcomes. The pathogenesis of T2 inflammation is characterized by a sophisticated interplay between the innate and adaptive immune systems. The process is often initiated at the mucosal barrier, where environmental insults trigger the release of epithelial-derived alarmins, including Thymic Stromal Lymphopoietin (TSLP), Interleukin-33 (IL-33), and Interleukin-25 (IL-25). These cytokines function as critical first responders that activate ILC2s (1). ILC2s are potent, antigen-independent sources of IL-5 and IL-13, playing a pivotal role in the early amplification of the eosinophilic response. By incorporating both Th2-cell mediated adaptive immunity and ILC2-driven innate pathways, the T2 landscape emerges as a redundant network where IL-5 serves as the terminal obligatory signal for eosinophil maturation, survival and activation. This detailed immunopathological framework provides the biological rationale for using high-affinity IL-5 inhibitors like mepolizumab, benralizumab and depemokimab to achieve profound and sustained eosinophil depletion.

#### 1.1.1 The “united airways” and systemic context

T2 inflammation is a systemic process that transcends individual organ involvement, often described through the united airways concept. This framework identifies the upper and lower respiratory tracts as a single immunological unit, where shared mediators drive conditions like SEA and CRSwNP (1). As discussed in section 1.1 regarding the role of epithelial alarmins and ILC2s, this cascade is not localized; it frequently involves systemic comorbidities such as atopic dermatitis or eosinophilic esophagitis. Understanding this systemic nature is crucial for the therapeutic positioning of long-acting biologics like depemokimab, which aim to stabilize the entire T2 axis.

### 1.2 Severe eosinophilic asthma and the glucocorticoid imperative

SEA affects approximately 5–10% of the adult asthmatic population, yet it accounts for a disproportionately large share of healthcare resource utilization and mortality (4). The eosinophilic phenotype is characterized by frequent and sometimes life-threatening exacerbations, persistent airflow limitation and reliance on systemic glucocorticoids (SGCs) to maintain symptomatic control (4). Chronic or repeated use of SGCs is associated with severe, dose-dependent adverse effects, including diabetes, osteoporosis, hypertension, cataracts and immune suppression, underscoring the imperative for effective steroid-sparing therapies (5). The advent of targeted biologics has been essential in reducing the dependency on SGCs while maintaining or even improving disease control and progression.

#### 1.2.1 Epidemiology and the Italian SANI perspective

In the specific context of severe asthma, eosinophilic phenotypes are the most prevalent, often characterized by a high burden of oral corticosteroids (OCS) use. Data from the SANI (Severe Asthma Network in Italy) (5) registry confirm that a significant proportion of Italian patients, up to 60% in real world cohorts, remain OCS-dependent, facing substantial risks of long-term systemic toxicity. This epidemiological reality underscores the importance of the steroid-sparing objective.

### 1.3 Chronic rhinosinusitis with nasal polyps

The patient population is further complicated by the high prevalence of CRSwNP, a T2-driven condition characterized by chronic inflammation and benign mucosal protrusions (polyps) within the nasal cavity and sinuses (2). CRSwNP causes debilitating symptoms such as severe nasal obstruction, loss of smell (anosmia), facial pain and chronic discharge, significantly impairing daily function. A substantial overlap exists: 25–60% of patients with SEA also suffer from CRSwNP, highlighting the systemic nature of T2 inflammation, the united airways concept and the need for treatments effective across both upper and lower airways (3). For patients with uncontrolled CRSwNP, who often face recurrent disease after functional endoscopic sinus surgery (FESS), targeted biologic therapy represents a crucial non-surgical intervention (2).

### 1.4 IL-5 as a therapeutic locus

Among the T2 cytokines, IL-5 holds a unique position as the principal and dedicated mediator of eosinophil biology. It controls the proliferation, maturation, trafficking and survival of eosinophils in the bone marrow and peripheral tissues (6–10). By neutralizing IL-5 or its receptor, biologic therapies aim to achieve profound and sustained eosinophil depletion, thereby interrupting the central inflammatory cascade in eosinophil-driven diseases in both the lung and sinonasal mucosa.

### 1.5 Immunological rationale for IL-5 blockade in systemic eosinophilic syndromes

The relevance of the IL-5/eosinophil axis extends beyond the respiratory tract, encompassing a spectrum of systemic disorders. In conditions like Eosinophilic Granulomatosis with Polyangiitis (EGPA) and Hypereosinophilic Syndrome (HES), the homeostatic control of eosinophils is lost, leading to systemic inflammation and tissue damage. From an immunological perspective, these diseases share the same IL-5-dependent effector mechanism. Therefore, the development of depemokimab represents a strategic approach to maintain continuous suppression of this pathway. Ongoing Phase III trials, such as NCT05263934 (EGPA) and NCT05334368 (HES), are currently evaluating whether the sustained pharmacological silencing of IL-5 can prevent systemic flares and reduce the cumulative burden of glucocorticoid-induced immunosuppression.

### 1.6 Therapeutic goals: from control to clinical remission

The management of T2 diseases is shifting from episodic symptom control to the achievement of clinical remission. In severe asthma, this state is defined by a composite of four criteria: 1) total absence of exacerbations, 2) elimination of maintenance OCS, 3) stabilization of lung function and 4) optimal symptom control (ACT >20) (11). For CRSwNP, the goal similarly extends beyond endoscopic scores (NPS); it focuses on clinically meaningful remission, including a significant reduction in SNOT-22 scores, restoration of smell and the avoidance of recurrent sinonasal surgery (12). These parameters provide the framework for evaluating the long term efficacy of depemokimab, particularly its potential to maintain a state of remission through sustained IL-5 suppression.

## 2 Methodology

To ensure a comprehensive and objective overview of the current landscape of IL-5 inhibition and the specific role of depemokimab, a structured literature search was performed.

### 2.1 Search strategy and data sources

We conducted a non-systematic electronic search across PubMed/MEDLINE, Embase and the Cochrane Central Register of Controlled Trials (CENTRAL). Furthermore, we consulted ClinicalTrials.gov to identify ongoing and recently completed Phase III programs. The search period included all relevant articles published from database inception up to December 2025, with a particular focus on the last five years to capture the most recent pharmacological developments.

### 2.2 Keywords and selection criteria

The search was performed using a combination of MeSH terms and keywords, including: *“depemokimab”*, *“GSK3511294”*, *“interleukin-5”*, *“IL-5 inhibition”*, *“eosinophilic asthma”*, *“CRSwNP”*, *“long-acting monoclonal antibodies”* and *“Type 2 inflammation”*.

Only articles published in English were considered. We prioritized:

1. Randomized controlled trials (RCTs) and their *post-hoc* analyses.
2. Pharmacokinetic and pharmacodynamic modeling studies.
3. Updated international guidelines (GINA, EPOS).
4. Peer-reviewed reviews discussing the role of ILC2s and epithelial alarmins in T2-high diseases.

Two independent authors (A.M.M. and E.B.) screened titles and abstracts for relevance. Discrepancies were resolved through discussion with a third senior author (A.G.). This approach was designed to provide a balanced synthesis of the pharmacological rationale and clinical potential of ultra-long-acting IL-5 blockade.

### 2.3 Research results

The search identified a total of 32 sources, including 29 peer-reviewed publications (original research, reviews and guidelines) and 3 clinical trial protocols from ClinicalTrials.gov representing ongoing or recently completed Phase III programs.

## 3 Depemokimab: mechanism of action and ultra-long pharmacokinetics

### 3.1 Mechanism of action

Depemokimab is a human Immunoglobulin G1 (IgG1) monoclonal antibody specifically directed against the IL-5 ligand. The goal of this targeted approach is to functionally remove IL-5 from circulation, preventing it from binding to the IL-5 receptor alpha chain (IL-5Rα) expressed on the surface of eosinophils, basophils, mastocytes and several other cell populations (10). This blockade suppresses the downstream biological activity of IL-5, leading to a profound dose-dependent and sustained suppression of eosinophils in the blood and affected tissues (11). The mechanism is highly selective, targeting only the eosinophilic component of the T2 inflammatory response. Practical considerations for the clinical use of depemokimab center on its simplified dosing schedule. The medication is administered as a 100 mg subcutaneous injection once every six months. To support different healthcare settings and patient preferences, it is designed for delivery via a pre-filled syringe or an autoinjector. This twice-yearly regimen is a significant departure from existing biologics and aims to resolve the persistent challenge of patient adherence in chronic respiratory care by reducing the frequency of interventions.

### 3.2 Pharmacokinetic/Pharmacodynamic profile and steady state

The pharmacodynamic profile of depemokimab is characterized by a rapid onset of action followed by a prolonged duration of effect (10). Following the first 100 mg subcutaneous injection, therapeutic plasma concentrations are achieved quickly, with the maximum concentration (Cmax) typically reached within 4 to 10 days. Due to the extended half-life provided by the YTE mutation, steady-state levels are generally attained by the time of the second dose at week 26. The PK/PD relationship is robust: the sustained plasma concentration remains well above the threshold required for near-complete neutralization of circulating IL-5, ensuring that the biological target is continuously inhibited throughout the six-month window.

### 3.3 The YTE modification and FcRn recycling

**Figure 1:**
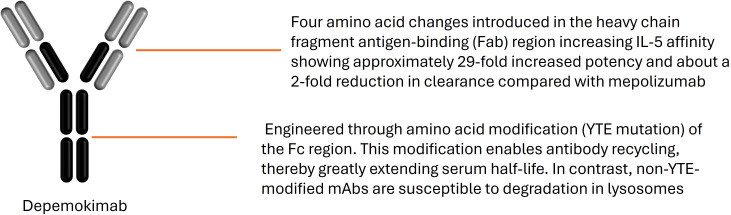
Molecular structure and YTE engineering of depemokimab. Depemokimab is a humanized IgG1 monoclonal antibody targeting Interleukin-5 (IL-5). The diagram highlights the two functional domains: the Fab region, responsible for high-affinity binding to soluble IL-5, and the engineered Fc region. The triple amino acid substitution (M252Y/S254T/T256E), known as the YTE modification, increases the binding affinity for the neonatal Fc receptor (FcRn) at acidic pH, promoting endosomal recycling and extending the systemic half-life to approximately 20 weeks.

The critical differentiator of depemokimab is its ultra-long-acting PK profile, which permits a twice-yearly dosing interval. This profile is engineered through a patented amino acid modification known as the YTE mutation (substitutions M252Y/S254T/T256E) within the Fc region of the antibody (10, 11) (**Figure 1**). This modification involves the substitution of three amino acids, methionine by tyrosine, serine by threonine, and threonine by glutamate, at positions 252, 254, and 256 of the IgG1 heavy chain. The primary objective of these changes is to exploit the natural recycling mechanism of the body. Typically, antibodies are taken up by cells and often degraded. However, the YTE mutation increases the strength of the bond between the antibody and the neonatal Fc receptor (FcRn) specifically in the acidic conditions of the intracellular endosome. Instead of being destroyed, the antibody is redirected back to the cell surface and released into the blood. This enhanced recycling loop extends the terminal half-life of depemokimab to approximately six months, which is nearly five times longer than standard monoclonal antibodies targeting the same pathway.

**Figure 2:**
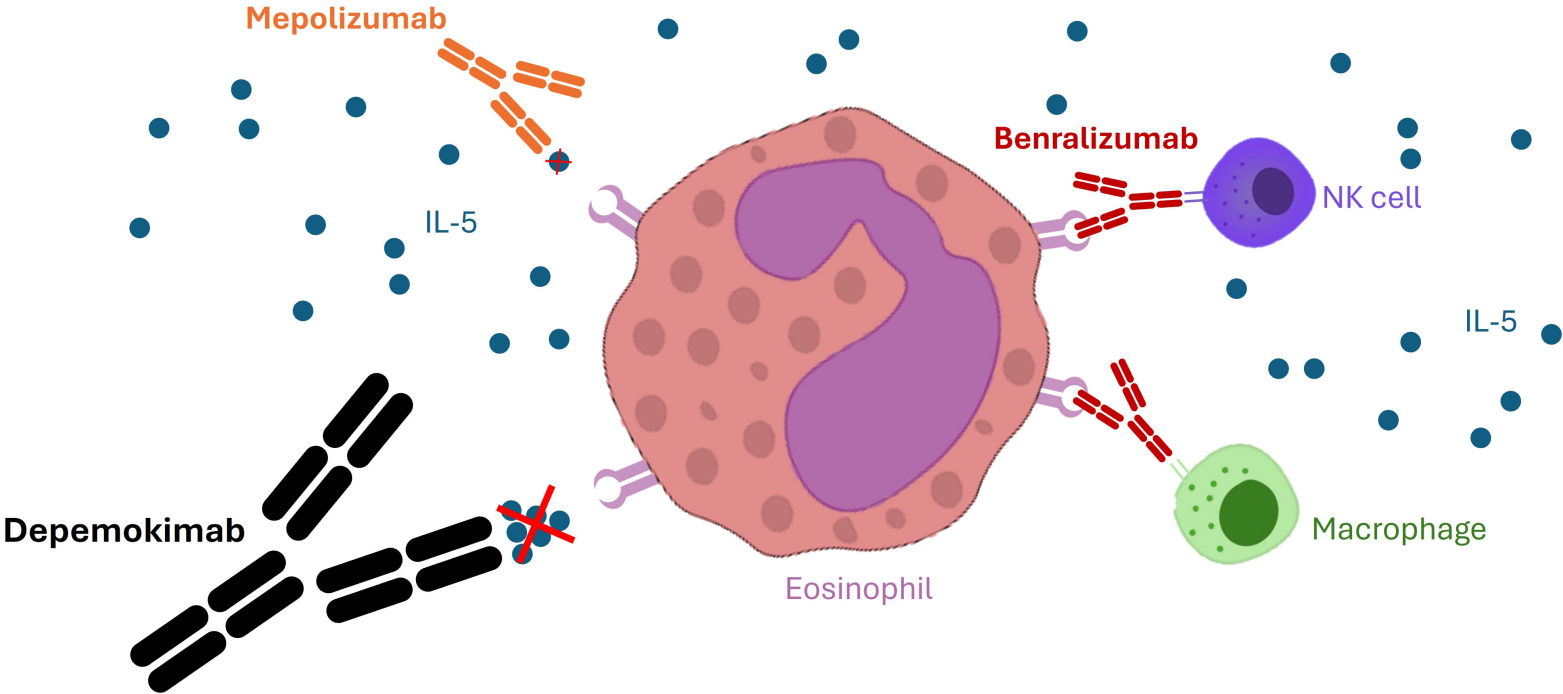
Molecular interactions and binding targets of anti-IL-5 pathway biologics. The figure illustrates the different pharmacological approaches to Interleukin-5 (IL-5) inhibition. While mepolizumab and depemokimab bind directly to the IL-5 cytokine, preventing its interaction with the alpha subunit of the IL-5 receptor (IL-5Rα), benralizumab targets the IL-5Rα on the surface of eosinophils and basophils, leading to antibody-dependent cell-mediated cytotoxicity (ADCC). Depemokimab’s high-affinity binding to soluble IL-5, combined with its engineered Fc-region (YTE modification), ensures prolonged neutralization of the T2-inflammatory cascade compared to first-generation anti-IL-5 agents.

The FcRn is integral to protecting IgG from lysosomal degradation by facilitating its recycling back to the circulation. By maximizing FcRn binding, the YTE modification ensures that a significantly higher proportion of depemokimab is rescued from lysosomal degradation, thus minimizing its clearance and drastically extending its serum half-life (11, 13). The resulting half-life of approximately 20 weeks robustly supports the Q6M (26-week) administration schedule, representing a significant PK breakthrough that sets it apart from all other approved biologics in respiratory disease (13). This mechanism ensures that effective therapeutic concentrations are maintained throughout the entire half-year interval. Four amino acid changes introduced in the heavy chain fragment antigen-binding (Fab) region increase IL-5 affinity, showing an approximately 29-fold increased potency and about a 2-fold reduction in clearance compared with mepolizumab (14) (**Figure 2**).

### 3.4 The 52-week eosinophil trajectory

The impact on effector cells is both immediate and persistent. Data from the SWIFT and ANCHOR programs show that blood eosinophil counts drop significantly by approximately 85-90% from baseline within the first 4 weeks of treatment. This suppression is maintained with high stability; the time course narrative over 52 weeks reveals a flat trajectory where eosinophil levels remain consistently below 50 cells/µL. Notably, there is no significant rise in eosinophil counts toward the end of the 26-week dosing cycle, confirming that the recycling mechanism of the YTE-modified antibody prevents the sub-therapeutic troughs often seen with shorter-acting biologics. This stability is central to the clinical efficacy of the regimen, as it provides uninterrupted protection against the eosinophilic surges that trigger exacerbations and polyp regrowth (8, 9, 14).

## 4 Clinical efficacy in severe eosinophilic asthma

The efficacy and safety of depemokimab in SEA were confirmed in two identical, multicenter, Phase III, randomized, double-blind, placebo-controlled, replicate trials: SWIFT-1 (n=395) and SWIFT-2 (n=397) (7).

### 4.1 Baseline characteristics

The efficacy of depemokimab was primarily established through the SWIFT-1 and SWIFT-2 phase 3 trials. The study population was representative of severe eosinophilic asthma: participants had a mean age of 52 years, a negligible smoking history (less than 10 pack-years), and high markers of T2 inflammation, with median baseline blood eosinophil counts of 360 cells/µL. Notably, approximately 15% of the cohort was receiving maintenance OCS at entry. A critical feature of this population was the high disease burden, evidenced by a mean of 2.1 clinically significant exacerbations in the year prior to screening, which served as the primary benchmark for assessing therapeutic impact.

### 4.2 Efficacy and subgroup analysis

In the pooled analysis of these trials, depemokimab achieved a 54% reduction in the annualized rate of clinically significant exacerbations compared to placebo (Rate Ratio 0.46; 95% CI 0.36–0.58). In practical terms, this represents a shift from an expected 1.08 exacerbations per year in the placebo arm to 0.46 in the depemokimab arm over the 52-week period. This clinical benefit remained consistent across various patient profiles. Specifically, the reduction in exacerbations was observed regardless of the baseline eosinophil count (above or below 300 cells/µL), the requirement for maintenance OCS at baseline, or whether patients had previously been treated with other biologics such as mepolizumab or benralizumab. These findings suggest that the six-month dosing interval provides sustained suppression of eosinophilic activity sufficient to stabilize the airway across different severity strata (8). As detailed in **Table 1**, depemokimab demonstrated a consistent and clinically significant reduction in the annualized rate of exacerbations across both SWIFT-1 and SWIFT-2 trials compared to placebo (p<0.001). Notably, the pooled analysis showed an even more pronounced effect on severe exacerbations requiring hospitalization or emergency room visits, with a 72% reduction (95% CI: 43–86%), highlighting the drug’s impact on the most resource consuming and intensive aspects of asthma care.

**Table 1:** Summary of key efficacy outcomes from phase III Depemokimab trials (SWIFT and ANCHOR).

Trial	Primary endpoint	Depemokimab group (result)	Placebo group (result)	Effect size (95% CI); p-value
SWIFT-1 (SEA)	Annualized rate of moderate/severe exacerbations	0.46	1.00	54% reduction (38–66%); p<0.001
SWIFT-2 (SEA)	Annualized rate of moderate/severe exacerbations	0.54	1.17	54% reduction (40–65%); p<0.001
SWIFT 1 + 2 (Pooled)	Rate of exacerbations requiring hospitalization/ER	0.05	0.18	72% reduction (43–86%); p<0.001
ANCHOR-1 (CRSwNP)	Change in Total Endoscopic Nasal Polyp Score (wk 52)	-1.5 (SE 0.2)	-0.3 (SE 0.2)	Diff: -1.2 (-1.7, -0.7); p<0.001
ANCHOR-1 (CRSwNP)	Change in Mean Nasal Obstruction Score (wk 49-52)	-0.90 (SE 0.08)	-0.33 (SE 0.08)	Diff: -0.57 (-0.79, -0.35); p<0.001
ANCHOR-2 (CRSwNP)	Change in Total Endoscopic Nasal Polyp Score (wk 52)	-1.7 (SE 0.2)	-0.2 (SE 0.2)	Diff: -1.5 (-2.0, -1.0); p<0.001
ANCHOR-2 (CRSwNP)	Change in Mean Nasal Obstruction Score (wk 49-52)	-1.02 (SE 0.08)	-0.31 (SE 0.08)	Diff: -0.71 (-0.93, -0.49); p<0.001

Clinical development program and efficacy outcomes of depemokimab in Type 2-mediated airway diseases. This table summarizes key data from the pivotal Phase 3 clinical trials, including the SWIFT and NIMBLE programs for SEA and the ANCHOR studies for CRSwNP. The reported outcomes emphasize the transition from traditional symptom control to the achievement of clinical remission, defined by the composite of zero exacerbations, elimination of maintenance oral corticosteroids (OCS), stabilization of lung function (FEV1), and optimal symptom control (ACT ≥20). For CRSwNP, efficacy is measured through clinically meaningful improvements in NPS and patient-reported outcomes via the SNOT-22 questionnaire, with a focus on restoration of olfaction and reduction in the need for recurrent surgery. Legend: SEA, Severe Eosinophilic Asthma; CRSwNP, Chronic Rhinosinusitis with Nasal Polyps; CI, Confidence Interval; SE, Standard Error.

### 4.3 Primary efficacy endpoint: exacerbation rate

The primary endpoint for both SWIFT trials was the Annualized Asthma Exacerbation Rate (AERR) (**Table 1**) (8). This outcome is highly relevant in SEA as exacerbations are the main driver of morbidity, SGC use and related healthcare costs.

In light of the pooled analysis conducted across the replicate SWIFT Phase III trials, depemokimab demonstrated a remarkably potent clinical impact on the primary endpoint of disease control. The therapeutic intervention resulted in a statistically significant and clinically robust reduction in the AERR. This substantial reduction is particularly noteworthy as it underscores the drug’s efficacy in stabilizing the underlying inflammatory process that leads to acute clinical worsening.

The data also reveals that depemokimab is equally effective in the most difficult-to-treat populations, including those with more severe disease manifestations who were already dependent on high-dose ICS to maintain even minimal control. By delivering a sustained and significant reduction in acute events regardless of these baseline complexities, the evidence from the SWIFT program establishes depemokimab as a highly effective agent for the long-term prevention of acute asthma events, positioning it as a possible cornerstone for future maintenance strategies in severe eosinophilic asthma.

### 4.4 Secondary efficacy endpoints: lung function and quality of Life

A critical analysis of the secondary outcomes within the SWIFT clinical program reveals a more complex and nuanced therapeutic profile for depemokimab compared to its primary success in exacerbation reduction. While the drug is effective in preventing acute events, the data regarding lung function, specifically measured by the Forced Expiratory Volume in 1 second (FEV1), showed no statistically significant differences between the depemokimab and placebo cohorts after 52 weeks of treatment. This suggests that depemokimab has a limited impact on chronic airway remodeling or the baseline airflow limitation that many patients with severe asthma face daily. Specifically, regarding lung function, the SWIFT trials reported a modest improvement in pre-bronchodilator FEV1, with mean increases ranging from 54 mL to 68 mL compared to placebo. While statistically significant in some analyses, these values are numerically lower than those historically observed with other anti-IL-5 agents such as mepolizumab (~100–120 mL) or benralizumab (~150 mL in specific subpopulations) (8). This discrepancy warrants an immunological and clinical reflection. It is important to note that the SWIFT study population had relatively high baseline FEV1 values (>60% predicted), which may have limited the margin for significant functional recovery (ceiling effect). Furthermore, these results reinforce the immunological concept that IL-5 inhibition primarily targets the eosinophilic-driven flare component of the disease rather than the structural airway remodeling or the smooth muscle hyperreactivity that predominantly dictate FEV1 values.

This stands in distinct contrast to biologic agents that inhibit the IL-4 and IL-13 pathways, which are more frequently associated with observable improvements in lung function.

Furthermore, this pattern of efficacy extended into the realm of Patient-Reported Outcomes (PROs) and overall Quality of Life (QoL). Throughout the study duration, authors observed no significant improvements in key subjective measures, including standard asthma symptom scores or the Asthma Quality of Life Questionnaire (AQLQ). This suggests that while the drug is powerful at preventing exacerbations, it may not substantially alter the daily symptomatic burden for some patients.

The clinical implication of these findings is that the value proposition of depemokimab is specifically anchored to two pillars: its ability to prevent exacerbations and its twice-yearly dosing frequency. Consequently, this therapy is most strategically positioned for patients whose primary clinical priority is the avoidance of acute asthma events and the reduction of SGC reliance. It may be less suitable as a first-line choice for patients whose main therapeutic goal is a significant improvement in daily symptom control or a substantial increase in baseline respiratory capacity. The decision to initiate depemokimab should therefore be guided primarily by the need for exacerbation control and treatment adherence rather than an immediate expectation of large scale lung function reversal. In patients where fixed airflow obstruction is the dominant clinical feature, the combination of sustained IL-5 blockade with optimized long-acting bronchodilators remains essential.

## 5 Efficacy in CRSwNP

The efficacy and safety in CRSwNP were evaluated in two replicate Phase III trials, ANCHOR-1 and ANCHOR-2 (9). This dual indication potential is crucial for patients with the interconnected T2 disease phenotype.

### 5.1 CRSwNP study design and outcomes

The Phase III clinical program for depemokimab expanded its investigation into the upper respiratory tract through the replicate ANCHOR-1 and ANCHOR-2 trials. These studies were specifically designed to include a broad and clinically challenging population, enrolling patients suffering from recurrent or uncontrolled CRSwNP, many of whom had already undergone sinus surgery without achieving long-term remission. To maintain consistency and evaluate the drug’s long-acting potential, these trials utilized the exact same Q6M dosing schedule over a 52-week period, directly comparing its performance against a placebo group.

The primary evaluation criteria for these trials were strategically selected to provide a comprehensive view of the drug’s efficacy, focusing on both the physical resolution of the disease and the relief of the patient’s daily burden. This was achieved through two critical, validated primary endpoints:

1. Nasal Polyp Score (NPS): This serves as the cornerstone for objective assessment. It is a validated scoring system where clinicians use endoscopic visualization to physically measure the size and extent of polyp growth within the nasal passages. By documenting a reduction in NPS, the ANCHOR trials were able to demonstrate depemokimab’s ability to physically shrink the inflammatory tissue causing the obstruction.
2. Nasal Congestion Score (NCS): While the NPS provides the biological proof of improvement, the NCS captures patients’ experience of the disease. As a key PRO, it requires patients to daily assess and record the severity of their nasal obstruction. This subjective metric is crucial, as it directly reflects the level of symptomatic improvement and the restoration of normal nasal breathing that patients prioritize.

The clinical implication of achieving success across both these endpoints is relevant. It demonstrates that depemokimab’s inhibition of IL-5, and the subsequent depletion of tissue eosinophils, is sufficient to not only reduce the physical mass of the polyps but also to significantly alleviate the persistent, suffocating sensation of congestion that defines the lives of those with severe CRSwNP. Such results confirm that the ultra-long-acting profile of depemokimab remains biologically active and therapeutically effective within the specialized environment of the sinonasal mucosa for the entire six-month dosing interval.

### 5.2 Baseline characteristics of the ANCHOR population

The enrolled population reflected a recalcitrant disease profile; nearly 75% of participants had undergone at least one prior sinonasal surgery (FESS), and approximately 25% had a history of three or more surgical interventions. At baseline, all patients were symptomatic despite the standard-of-care use of intranasal corticosteroids. The severity of the cohort was further underlined by high baseline scores, with a mean endoscopic NPS of 5.7 out of 8 and a NCS of approximately 2.3 out of 3, indicating significant bilateral obstruction and impaired quality of life.

### 5.3 Efficacy findings

In the ANCHOR-1 and ANCHOR-2 trials, depemokimab met both co-primary endpoints. The 52-week results from the ANCHOR program showed substantial and sustained clinical improvements with twice-yearly depemokimab. In ANCHOR-1 and ANCHOR-2, the treatment group showed a mean reduction in NPS of -1.8 and -1.9 points respectively, compared to -0.6 and -0.8 in the placebo groups. Similarly, the NCS improved by an average of -1.1 points from baseline in the depemokimab arms, nearly doubling the improvement seen with placebo (-0.6). These absolute changes were accompanied by a significant reduction in the need for systemic corticosteroid rescue courses and a lower rate of nasal surgery during the follow-up period. These data confirm that sustained IL-5 inhibition, even with extended dosing intervals, effectively reduces the inflammatory mass and restores sinonasal patency in a population characterized by previous treatment failures.

The improvement in the total endoscopic NPS and the significant reduction in nasal obstruction scores (**Table 1**) underscore the efficacy of sustained IL-5 suppression in remodeling upper airway tissues, even with a twice-yearly dosing interval.

The data demonstrated that treatment with depemokimab led to a substantial and sustained decrease in the physical size of nasal polyps when compared to the placebo group. This is a critical observation because it confirms an objective anatomical improvement; it proves that the high-affinity binding to IL-5 and subsequent eosinophil depletion actually results in the shrinkage of the inflammatory tissue masses that obstruct the nasal passages (9).

The second and equally vital result was the NCS reduction. Beyond the clinical measurements, patients reported a significant decrease in nasal obstruction. This subjective improvement is relevant, as congestion is consistently cited by patients as the most debilitating symptom of CRSwNP, affecting everything from sleep quality to daily cognitive focus (9).

These results support the therapeutic role of anti-IL-5 therapy when delivered via an ultra-long-acting regimen, demonstrating that the T2 inflammatory burden across the upper airway can be effectively managed even with a vastly extended dosing interval. For the CRSwNP patient community, the ability to achieve both a physical reduction in polyp volume and a subjective relief from chronic congestion with only two injections per year may represent an advantage. This simplicity is particularly valuable in a disease landscape characterized by high recurrence rates and the constant, exhausting cycle of frequent surgical procedures or repeated office-based interventions.

## 6 Safety profile

### 6.1 Safety and tolerability

The safety profile of depemokimab has been comprehensively evaluated throughout the Phase III clinical development program, spanning both the SWIFT and ANCHOR trials to ensure a robust understanding of its risk-benefit ratio across different respiratory indications. Overall, depemokimab demonstrated a highly favorable tolerability profile, with the total incidence of treatment-emergent adverse events (TEAEs) remaining remarkably comparable between the active treatment arms and the placebo cohorts in both SEA and CRSwNP (8, 9). This lack of divergence is a critical finding, as it suggests that the ultra-long-acting nature of the molecule does not introduce any novel or unexpected safety signals that differ from existing, shorter-acting anti-IL-5 therapies.

When examining the severity of these events, the data regarding Serious Adverse Events (SAEs) are reassuring, showing an incidence rate that was both low and statistically similar to that of the placebo. This indicates that the potent and near-complete depletion of eosinophils maintained by depemokimab does not compromise essential immune functions or increase the risk of severe systemic complications.

Furthermore, the most common TEAEs observed were typical of the subcutaneous biologic class and generally mild in nature. These primarily included transient injection site reactions, which were both mild-to-moderate and self-limiting, as well as headache and nasopharyngitis (8).

Perhaps the most significant indicator of the drug’s clinical acceptance is the extremely low rate of treatment discontinuations due to adverse events (8, 9). This high level of persistence over the full 52-week treatment period underscores depemokimab’s overall tolerability. For patients, this means that the transition to a twice-yearly dosing regimen does not come at the cost of increased side effects, supporting its role as a stable, long-term maintenance therapy for chronic T2-mediated airway diseases.

### 6.2 Immunogenicity and anti-drug antibodies

A critical technical consideration for any therapeutic protein designed for long-term administration, especially an ultra-long-acting biologic like depemokimab, is the potential for immunogenicity, which refers to the body’s immune system recognizing the drug as foreign and developing anti-drug antibodies (ADAs). From a pharmacological perspective, there is a legitimate theoretical concern that extended dosing intervals might increase this risk; specifically, maintaining low but detectable trough levels of the therapeutic protein for prolonged periods could potentially facilitate a sustained exposure to the immune system, thereby promoting the development of ADAs over time (8, 10, 13).

However, the empirical evidence from the Phase III clinical program provides a reassuring counter-narrative to these theoretical risks. The observed incidence of ADAs across the replicate studies was reported as notably low, with a rate of approximately 12% in the SWIFT-1 cohort and an even lower incidence of 5% in SWIFT-2 (8). These data suggest that the specific molecular engineering of depemokimab, including the YTE modification, does not inherently make the molecule highly immunogenic despite its long residence time in the systemic circulation (8, 15).

Crucially, the clinical impact of these antibodies appears to be negligible in the majority of cases. Detailed analysis revealed that the vast majority of patients who did test positive for ADAs continued to maintain effective eosinophil suppression, mirroring the results seen in the ADA-negative population. Furthermore, there was no discernible evidence that the presence of these antibodies negatively impacted the drug’s PK profile or its primary efficacy in reducing exacerbations (8, 15).

Despite this highly encouraging initial dataset, a cautious approach is still warranted. As the drug is intended for life-long maintenance therapy with only two exposures per year, ongoing long-term surveillance remains essential. Further observation through open-label extension studies and real-world evidence will be vital to fully assess the clinical significance of ADA development over multiple years of Q6M dosing, ensuring that the safety and efficacy profile remains stable as the patient’s immune system is repeatedly exposed to the drug over a long-term horizon.

### 6.3 Regional landscape and reslizumab

The current management of SEA and CRSwNP relies on a well-established set of biologics. It is important to note that the clinical landscape described herein reflects the Italian regulatory context. In Italy, the antieosinophil arsenal is currently composed of mepolizumab and benralizumab, as reslizumab is not available in the national formulary. Consequently, the transition to depemokimab represents a shift within a specific framework of subcutaneous therapies.

## 7 Comparative efficacy and existing treatment landscape

**Figure 3:**
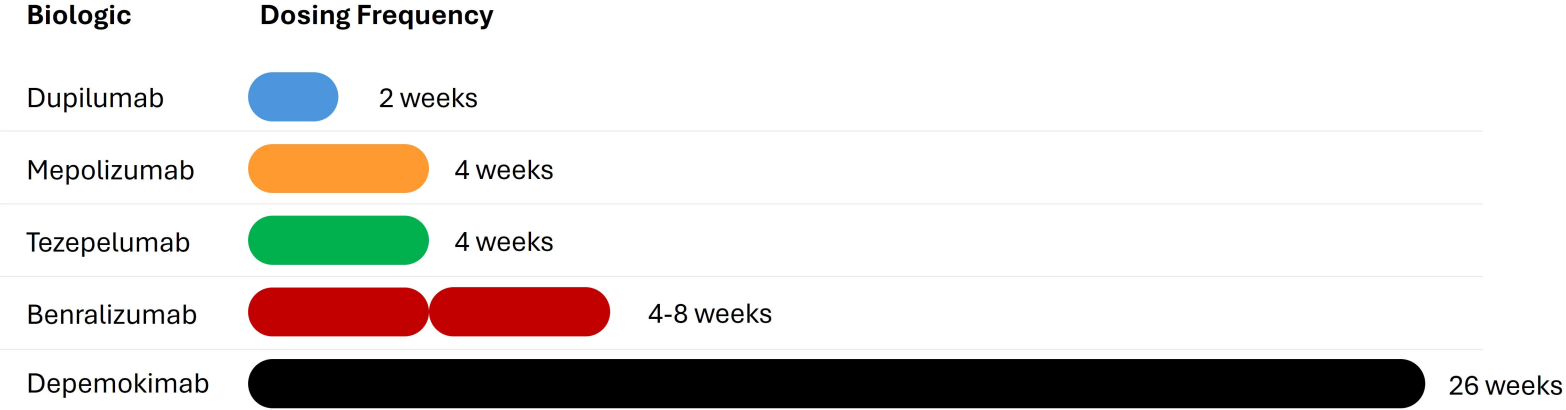
Comparison of dosing frequencies among IL-5 pathway inhibitors. The bar chart illustrates the significant extension of the therapeutic interval enabled by depemokimab compared to first-generation biologics. While mepolizumab, reslizumab, and benralizumab require administration every 4 to 8 weeks to maintain clinical efficacy, depemokimab’s ultra-long-acting profile supports a twice-yearly (Q6M) dosing regimen. This 24-week interval, facilitated by the YTE Fc-engineering, represents a paradigm shift in chronic disease management by potentially reducing treatment fatigue and improving long-term patient adherence.

Depemokimab enters an increasingly crowded market of T2 biologics. Its success will rely not only on its efficacy but also on its differentiated profile when compared to existing anti-IL-5 agents and inhibitors of other T2 pathways (**Figure 3**; **Table 2**).

**Table 2:** Comparative pharmacology of T2 biologics.

Feature	Depemokimab	Mepolizumab	Benralizumab	Dupilumab	Tezepelumab
Primary Target	IL 5 Ligand	IL 5 Ligand	IL 5R alpha	IL 4R alpha	TSLP
Dosage	100 mg	100 mg	30 mg	200 or 300 mg	210 mg
Dosing Frequency	Every 6 months	Every 4 weeks	Every 8 weeks*	Every 2 weeks	Every 4 weeks
Best Phenotype	High Eosinophils	High Eosinophils	High Eosinophils	Eos + FeNO	Broad T2
Immunogenicity	Low	Low	Low	Low	Low
Admin Route	SC (HC Prof)	SC (Self/HC Prof)	SC (Self/HC Prof)	SC (Self admin)	SC (Self/HC Prof)

Comparative pharmacological profiles and administration protocols of biological therapies for T2 inflammatory diseases. This table illustrates the distinct molecular targets and pharmacokinetic properties of current and emerging biologics. The comparative analysis underscores how phenotypic selection, relying on biomarkers such as eosinophil counts and fractional exhaled nitric oxide (FeNO), remains critical for optimizing treatment response across the T2-inflammatory spectrum, including SEA, CRSwNP and broader eosinophilic conditions. Legend: IL-5, Interleukin-5; IL-4Rα, Interleukin-4 Receptor alpha; TSLP, Thymic Stromal Lymphopoietin; T2, Type 2 inflammation; SEA, Severe Eosinophilic Asthma; CRSwNP, Chronic Rhinosinusitis with Nasal Polyps; EGPA, Eosinophilic Granulomatosis with Polyangiitis; HES, Hypereosinophilic Syndrome; EoE, Eosinophilic Esophagitis; FeNO, Fraction of exhaled Nitric Oxide; HC Prof., Healthcare Professional.

### 7.1 The anti-IL-5 class comparison

The therapeutic landscape for SEA has been defined by the established anti-IL-5 class, which has fundamentally changed the management of T2 inflammation. This class currently includes two primary biologic agents, each with a distinct molecular target and administration protocol:

- Mepolizumab: a humanized monoclonal antibody that targets the IL-5 ligand directly. It is typically administered subcutaneously once every 4 weeks (Q4W) (16).
- Benralizumab: this agent takes a different approach by targeting the IL-5 receptor alpha (IL-5Rα) on the surface of eosinophils, leading to their direct depletion via antibody-dependent cell-mediated cytotoxicity (ADCC). Its dosing schedule involves an initial induction phase (Q4W for the first three doses) followed by maintenance injections every 8 weeks (Q8W) (17).

From a clinical perspective, all these agents have demonstrated a remarkably consistent and comparable level of efficacy. They typically achieve a reduction in the AERR ranging between 40% and 60% compared to placebo, while simultaneously inducing profound eosinophil depletion in the blood. This consistent performance across the class has firmly validated the therapeutic strategy of IL-5 inhibition as a cornerstone for treating eosinophilic respiratory diseases. Similar results were obtained for another anti-IL-5 drug, reslizumab, administered intravenously but less widely used than the previous two (18).

However, while the clinical outcomes are similar, the logistical burden placed on the patient varies significantly. The traditional dosing schedules, ranging from monthly to bimonthly, contrast with the Q6M regimen of depemokimab. By requiring only two doses per year, depemokimab represents a 3-fold to 6-fold reduction in the total number of administrations required annually. This logistical difference also minimizes the need for frequent clinical visits, reduces the burden of illness for the patient and significantly simplifies long-term disease management compared to the more frequent injection cycles of other anti IL-5/IL-5Rec drugs.

When placing depemokimab within the current therapeutic hierarchy, a comparison with mepolizumab, the first-in-class anti-IL-5 biologic, is essential. In the pivotal MENSA (16) and DREAM (19) trials for severe eosinophilic asthma, mepolizumab 100 mg subcutaneous administered every 4 weeks demonstrated a reduction in the annualized rate of exacerbations ranging from 47% to 53%. Remarkably, the pooled data from the depemokimab SWIFT program show a nearly identical efficacy profile, with a 54% reduction in exacerbations (8).

Similarly, in the context of CRSwNP, the SYNAPSE trial (20) established the efficacy of monthly mepolizumab in reducing nasal polyp score and obstruction. The depemokimab ANCHOR trials (9) reported absolute improvements in these same endoscopic and clinical markers that align with the results seen in SYNAPSE. While cross-trial comparisons must be interpreted with caution due to differences in baseline characteristics and trial duration, these observations suggest that the YTE-modification does not sacrifice potency for longevity. The primary clinical differentiator is the optimization of the PK profile: depemokimab maintains a level of disease stability equivalent to monthly or bimonthly IL-5 inhibition, but achieves this with only two interventions per year. This shift addresses the “ceiling” of efficacy in IL-5 blockade while significantly lowering the cumulative treatment burden for the patient.

### 7.2 Clinical need and the adherence gap

The clinical positioning of depemokimab addresses a specific unmet need that persists even with the availability of effective agents like mepolizumab. While existing biologics have revolutionized T2 disease management, their requirement for frequent administration, ranging from every 4 to 8 weeks, introduces a significant treatment burden. Real-world evidence highlights an adherence gap where patient persistence declines over time due to logistical challenges or the psychological weight of chronic injections. Depemokimab, by virtue of its twice-yearly regimen, may be positioned to bridge this gap. This schedule aims to decouple biological efficacy from the frequency of intervention, ensuring that the bottleneck cytokine IL-5 remains suppressed continuously. This approach could prove effective, particularly for patients who struggle with the maintenance of shorter-interval therapies.

### 7.3 Comparison to other T2 biologics

When analyzing the therapeutic landscape, it is essential to compare depemokimab with biologics that intervene in different segments of the T2 inflammatory cascade. Two of the most prominent alternatives are dupilumab and tezepelumab, each offering a distinct clinical profile and dosing requirement:

- Dupilumab: this monoclonal antibody targets the IL-4Rα subunit, effectively blocking the signaling pathways of both IL-4 and IL-13. Typically administered Q2W (21), it is widely recognized for its multi-dimensional impact. Beyond its ability to reduce the AERR, dupilumab consistently delivers significant improvements in lung function (FEV1) and overall QoL scores in patients with severe eosinophilic asthma. In the context of nasal polyps, it shows a robust capacity to resolve complex symptoms, including the particularly challenging issue of anosmia (22).
- Tezepelumab: unlike agents that target specific downstream cytokines, tezepelumab acts upstream by blocking TSLP, an epithelial-derived alarmin. Dosed every four weeks (23), its primary advantage lies in its versatility, as it targets a cytokine released at the very start of the inflammatory response; it remains effective across a broader spectrum of asthma phenotypes, including cases with low eosinophil counts where other biologics might fail (23).

The mixed results observed for depemokimab regarding secondary endpoints, specifically the lack of significant FEV1 and QoL improvements seen in the SWIFT trials, create a clear differentiation from an agent like dupilumab. Consequently, the selection of the most appropriate biologic must be tailored to the patient’s dominant clinical phenotype and primary goals.

Dupilumab remains the preferred choice for patients who require comprehensive symptom relief and measurable gains in respiratory capacity. On the other hand, depemokimab is strategically positioned for the patient whose clinical management is defined by two priorities: the prevention of exacerbations and the desire to minimize the frequency of medical interventions through its twice-yearly dosing schedule.

### 7.4 Differential biologic selection in T2-high diseases

The therapeutic landscape of severe asthma has evolved from targeting single cytokines to broader pathway modulation. When comparing depemokimab with dupilumab (anti-IL-4Rα) and tezepelumab (anti-TSLP), the clinician must consider the dominant immunophenotype. Dupilumab is particularly effective in patients with coexisting T2 comorbidities, such as atopic dermatitis or eosinophilic esophagitis, where IL-4 and IL-13 play a more systemic role than IL-5 (24). Tezepelumab, by acting upstream on epithelial alarmins, offers a broader response in patients who may not reach the high eosinophil thresholds required for classic anti-IL-5 therapy (23). Depemokimab offers a precision strike against the eosinophil. For patients whose disease is strictly driven by eosinophilic infiltration, the ultra-long-acting inhibition of IL-5 may provide a more stable suppression of the primary effector cell. Regarding steroid-sparing effects, preliminary data from the SWIFT program suggest that depemokimab’s ability to reduce OCS reliance is comparable to the 50-70% reduction rates seen with dupilumab and mepolizumab, though long-term real-world data are still pending.

### 7.5 The pending non-inferiority data

The definitive positioning of depemokimab within international consensus guidelines and clinical treatment algorithms is currently in a transitional phase as the medical community awaits the highly anticipated results of the ongoing head-to-head non-inferiority trials. These studies are specifically designed to directly compare the clinical efficacy of depemokimab against the established standards of care in the anti-IL-5 class, namely mepolizumab and benralizumab (25). This evidence is crucial for determining whether the extended dosing interval maintains the same rigorous level of disease control offered by more frequent administrations.

If these comparative studies successfully confirm that depemokimab is non-inferior in its ability to suppress eosinophilic inflammation and prevent exacerbations, its PK profile will redefine the criteria for patient selection. In such a scenario, the Q6M dosing regimen immediately transitions from a secondary convenience feature to a compelling clinical advantage of the drug. By offering the same protective benefits as monthly or bimonthly biologics but with only two interventions per year, depemokimab would likely become the preferred choice for healthcare providers seeking to optimize long-term adherence and reduce the logistical burden on both patients and specialized respiratory clinics.

This shift would represent a fundamental change in the management of severe eosinophilic respiratory diseases, moving away from high-frequency maintenance and toward a more streamlined, long-term protection model that preserves the quality of life while ensuring maximum safety from acute events.

### 7.6 Biomarkers and precision immunology

In the era of precision immunology, understanding the diverse sources of IL-5 (both Th2 and non-Th2) is crucial for therapeutic selection. While upstream inhibitors targeting alarmins offer a broad immunomodulatory effect (23), direct and sustained IL-5 inhibition remains a highly targeted strategy for patients whose immunotype is predominantly eosinophilic (8, 9, 16–18). Depemokimab ensures a constant therapeutic pressure on the IL-5 receptor ligand, potentially overcoming the rebound eosinophilia sometimes observed with shorter-acting biologics, thus offering a more stable immunological control.

## 8 Patient-centricity, adherence and healthcare economics

The introduction of ultra-long-acting biologics such as depemokimab may represent a significant shift in the management of chronic T2 inflammatory diseases (25–27). Beyond the direct clinical outcomes of eosinophil depletion and exacerbation reduction, the value of a Q6M dosing regimen is rooted in its ability to align medical therapy with the practical realities of patient lives and the logistical constraints of healthcare systems (26). This transition from frequent administration to a biannual schedule directly addresses the fundamental logistical and psychological barriers inherent in managing chronic respiratory conditions (25–27).

### 8.1 The burden of frequent dosing and adherence

In chronic disease management, the complexity and frequency of a treatment schedule are among the most significant predictors of long-term adherence and patient persistence (26). For patients with SEA and CRSwNP, traditional biologic therapies require a commitment to frequent clinical interventions or self-injections, typically ranging from every 2 to 8 weeks (16–23). Suboptimal adherence to these frequent medication regimens remains a major clinical hurdle, inevitably leading to poor disease control, a higher frequency of acute events and increased healthcare costs (26). From a pharmacoeconomic perspective, for injectable biologics, the frequency of administration itself is a recognized barrier to consistent therapy, often resulting in sub-therapeutic coverage if doses are missed or delayed (26, 27).

Quantitative surveys of patients and healthcare providers consistently indicate a strong preference for less frequent dosing intervals and a reduction in the number of required clinic visits (27, 28). Depemokimab’s Q6M schedule stands out as the least frequent among all currently approved biologic agents for SEA and CRSwNP, directly addressing these patient-reported needs. Furthermore, frequent injections often serve as a constant reminder of the patient’s chronic condition, which significantly contributes to treatment fatigue and a perceived increase in the burden of disease (26–28). By requiring only two interventions per year, the Q6M schedule may minimize both the time burden and the psychological stress associated with frequent medicalization, eventually leading to better long-term persistence on therapy compared to more intensive regimens (27).

### 8.2 Healthcare system impact and resource optimization

The Q6M regimen offers advantages for health system management and the strategic allocation of clinical resources (28, 29). While most current biologics for respiratory diseases require administration every 2 to 8 weeks, reducing the frequency of in-office injections or monitoring visits substantially alleviates the logistical and administrative burden on specialty clinics, nursing staff and pharmacy departments (28). This increased clinical efficiency allows for a more sustainable management model, particularly in healthcare settings where nursing capacity is a limiting factor in biologic delivery (29).

Recent analyses of long-acting therapies across various immunomediated diseases suggest that reduced administration frequency can alleviate healthcare bottlenecks, improving the throughput of specialized clinics without requiring additional personnel (29). When considering the economic drivers of severe T2 disease, it is clear that while the acquisition cost of biologic therapy is a major factor, the total economic burden is primarily driven by acute events necessitating emergency care and hospitalizations (16–27). By combining a convenient schedule that promotes adherence with a powerful anti-exacerbation effect, including a 54% reduction in the AERR, depemokimab has the potential to translate clinical efficacy into substantial long-term cost savings by preventing the most expensive manifestations of the disease.

### 8.3 Strategic positioning: efficacy meets adherence

**Figure 4:**
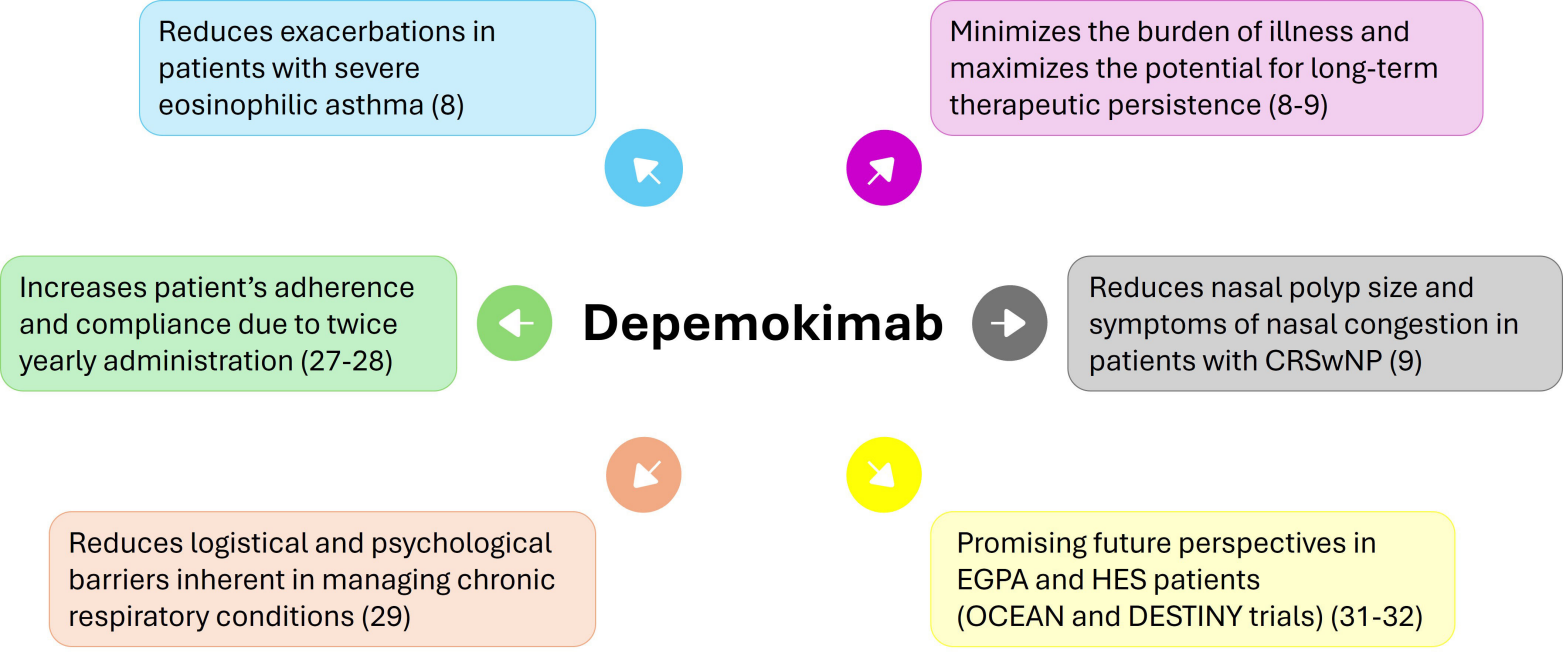
Therapeutic positioning of depemokimab in the management of severe airway diseases. The diagram illustrates the clinical placement of depemokimab within the treatment algorithms for severe eosinophilic asthma (SEA) and chronic rhinosinusitis with nasal polyps (CRSwNP). Depemokimab is positioned as a strategic option for patients with a confirmed Type 2-high phenotype who may benefit from an ultra-long-acting biological therapy. This positioning considers both clinical/laboratory markers (blood eosinophil counts, exacerbation history, and nasal polyp burden) and patient-related factors, such as the need to optimize adherence and reduce the treatment burden associated with more frequent injection schedules.

The strategic value proposition of depemokimab lies in the integration of established anti-IL-5 efficacy with a simplified dosing protocol (7, 8, 12). While other biologics targeting different pathways may offer broader symptom relief or greater lung function gains in specific sub-populations, depemokimab’s role is best targeted to patients where exacerbation control and minimizing injection frequency are the clinical priorities. For individuals with a history of poor adherence or those who face significant geographical or logistical barriers to frequent clinic visits, the Q6M schedule offers a pharmacological solution that minimizes the burden of illness and maximizes the potential for long-term therapeutic persistence (27). Ultimately, the shift toward ultra-long-acting agents reflects an evolving clinical focus on patient-centered outcomes and healthcare sustainability (28–30) (**Figure 4**). These characteristics may also be very useful in systemic diseases related to eosinophils and IL-5, such as EGPA and HES, for which clinical trials are currently underway.

### 8.4 OCS-sparing potential and SNOT-22 clarity

A key consideration for any biologic in severe asthma is its ability to reduce the burden of OCS. Although the SWIFT trials were primarily designed to assess exacerbation rates, the near-complete and uninterrupted suppression of IL-5 provides a strong pharmacological rationale for OCS-sparing effects (8). By maintaining eosinophils at near-zero levels for the entire six-month cycle, depemokimab may prevent the flare ups that typically necessitate rescue OCS. Regarding the CRSwNP data, it is worth noting that while the ANCHOR trials focused on the NPS and NCS as primary benchmarks, patient-reported outcomes like the SNOT-22 were considered secondary or exploratory (9). Future real-world evidence will be essential to fully characterize the impact of this long-acting regimen on the comprehensive quality-of-life domains captured by the SNOT-22.

### 8.5 Real-world management of a Q6M regimen

The transition to a twice-yearly dosing schedule introduces unique practical considerations. While the Q6M regimen significantly lowers the treatment burden and likely improves long-term persistence, it also alters the traditional rhythm of clinical monitoring. In a six months window, a patient’s clinical status, such as the development of new comorbidities or changes in environmental triggers, can evolve significantly. Therefore, the use of depemokimab should be accompanied by robust inter-visit management strategies. This may include digital health tools for remote symptom tracking or clear red flag protocols to ensure that patients remain connected to their specialist even when the frequency of in-office injections is reduced.

## 9 Limitations of the current evidence base

While the Phase III clinical data provide a robust foundation for the efficacy of depemokimab, several limitations inherent in the current evidence base necessitate a cautious interpretation regarding its broad clinical application.

The first significant constraint involves the mixed secondary endpoint profile observed in the SWIFT and ANCHOR trials. Unlike other biologics such as dupilumab, which have consistently demonstrated significant improvements in FEV1 and St. George’s Respiratory Questionnaire (SGRQ) scores, depemokimab did not achieve statistically significant superiority in these measures over placebo in all trial cohorts (8). This limitation restricts its potential utility in patients for whom daily symptom control and measurable gains in lung function are the primary therapeutic objectives, but a nuanced interpretation is required when evaluating the impact of depemokimab on lung function. While some biologics initially aimed for significant increases in FEV1, it is increasingly recognized that in cohorts with long-standing disease and structural airway remodeling, the primary goal is the preservation of existing function. This perspective is aligned with the clinical remission criteria proposed by the Severe Asthma Network Italy (SANI) (5), which define functional remission as the stability of lung function rather than a mandatory numerical increase. In the SWIFT trials, the ability of depemokimab to maintain stable FEV1 values over 52 weeks while simultaneously halving exacerbation rates should be viewed as a key component of clinical stabilization (8). This suggests that sustained IL-5 inhibition effectively mitigates the inflammatory surges that drive progressive loss of lung function, even if the baseline fixed obstruction remains unchanged. Furthermore, the duration of follow-up in the current dataset is relatively short. The core efficacy and safety data are primarily derived from 52-week trials (8, 9). Consequently, long-term multi-year safety profiles, particularly regarding the impact of low but detectable ADA and immunogenicity, remain to be fully elucidated. Real-world persistence and the maintenance of eosinophil suppression over several years require ongoing pharmacovigilance and long-term extension studies to confirm that depemokimab’s regimen does not lead to “escape” inflammation toward the end of the dosing cycle.

The absence of head-to-head comparative data also represents a critical gap in the current literature. Although cross-trial comparisons suggest comparable reductions in the AERR to existing agents like mepolizumab and benralizumab, definitive conclusions regarding relative efficacy and safety cannot be drawn without direct comparative trials. The medical community is currently awaiting the publication of planned non-inferiority studies designed to evaluate depemokimab against established Q4W and Q8W biologics (25).

Finally, the generalizability to diverse populations remains a work in progress. While the SWIFT and ANCHOR trials utilized global recruitment, clinical trial populations are often highly selected and may not reflect the complexity of real-world patients. Data are still needed to confirm efficacy across various ethnic backgrounds, extreme baseline disease severities and in patients with multiple significant comorbidities. Establishing performance metrics in diverse demographic groups is essential for ensuring that the logistical benefits of the Q6M regimen are realized equitably across the global patient population.

### 9.1 Beyond respiratory diseases: EGPA and HES

The therapeutic potential of depemokimab extends beyond the respiratory system, addressing systemic conditions where IL-5-driven eosinophilia plays a central pathogenic role. In EGPA and HES, sustained suppression of eosinophils is not only a matter of symptom control but a necessity to prevent irreversible organ damage. The immunological rationale for using an ultra-long-acting IL-5 inhibitor in these contexts lies in its ability to provide a molecular clamp on eosinophil production, avoiding the fluctuations that can occur with traditional therapies. Currently, the clinical development of depemokimab is being evaluated in two pivotal trials: the OCEAN study (NCT05263934) (31) for EGPA and the DESTINY study (NCT05334368) (32) for HES. These trials aim to demonstrate that the 6-month dosing interval can maintain clinical remission and reduce the cumulative exposure to high-dose corticosteroids, which remains a significant burden for these patients. By targeting the IL-5 bottleneck with high affinity and prolonged durability, depemokimab could redefine the management of systemic eosinophilic syndromes, shifting the goal from reactive treatment of flares to long-term preemptive stabilization.

## 10 Conclusion and future positioning

Depemokimab represents a transformative development in the therapeutic landscape of severe T2 inflammation, offering a pharmacological bridge between established anti-IL-5 efficacy and ultra-long-acting delivery. By leveraging the YTE aminoacid modification to enhance FcRn binding affinity, this agent successfully extends the duration of IL-5 inhibition, allowing for a Q6M dosing regimen that remains unique within the respiratory biologic space. The validation of this strategy through Phase III trials confirms that depemokimab provides a robust and clinically significant reduction in the AERR, approximately 54%, while simultaneously reducing the clinical burden in CRSwNP as measured by nasal polyp and congestion scores (8, 9).

The core value proposition of depemokimab lies in the integration of validated clinical efficacy, specifically targeted at the prevention of acute events, with a level of patient convenience and dosing simplicity that is unmatched by currently approved biologics (8, 16–21). Its strategic placement within the treatment algorithm is likely to be defined by a prioritization of patient lifestyle and adherence needs, especially for individuals who face significant logistical barriers, such as distance to specialized clinics, or those with a documented history of poor persistence with frequent medication schedules (**Tables 3**, **4**).

**Table 3:** Summary of clinical evidence and strategic positioning of depemokimab.

Domain	Key findings & clinical implications	Supporting evidence
Primary Efficacy	Robust reduction in Annualized Asthma Exacerbation Rate (AERR) by ~54% and significant decrease in nasal polyp burden (NPS/NCS).	SWIFT-1/2 (8), ANCHOR-1/2 (9)
Pharmacokinetics	YTE modification extends half-life by enhancing FcRn binding, enabling a twice-yearly (Q6M) dosing interval.	Robbie et al. (13), Sutton et al. (14)
Patient Adherence	Addresses “treatment fatigue” and logistical barriers; Q6M is the least frequent regimen currently available.	Foster et al. (26), Albers et al. (28)
Healthcare Impact	Potential for reduced administrative overhead and long-term savings by preventing acute, high-cost emergency events.	Sampson et al. (29), Moots et al. (30)
Secondary Endpoints	Inconsistent gains in lung function (FEV1) and Quality of Life (QoL) compared to daily-relief focused biologics.	Sutton et al. (8), Bachert et al. (9)
Evidence Gaps	Long-term multi-year safety, immunogenicity (ADA impact), and definitive head-to-head non-inferiority data.	SWIFT-1/2 (8), NIMBLE Trial (25)

Summary of the therapeutic profile and strategic value of depemokimab in severe Type 2 (T2) inflammatory diseases. The table highlights the integration of established anti-IL-5 efficacy with ultra-long-acting pharmacokinetics. While the Q6M regimen optimizes adherence and healthcare resource allocation, the evidence base currently shows mixed results for secondary functional endpoints (FEV1 and QoL), suggesting a specialized role for depemokimab in exacerbation-prone patients or those requiring a simplified treatment burden.

**Table 4:** Summary of clinical evidence, therapeutic positioning, and evidence gaps for depemokimab.

Domain	Key findings & clinical evidence	Strategic positioning & patient selection
Pharmacological Foundation	YTE amino acid modification enhances neonatal Fc receptor (FcRn) binding, extending half-life to enable Q6M dosing (10, 13, 14).	Dosing Simplification: Positions depemokimab as the primary choice for patients with significant geographical or logistical barriers to care.
Clinical Efficacy	~54% reduction in AERR in severe asthma; significant reduction in NPS and NCS in CRSwNP (8, 9).	Exacerbation-Dominant Phenotype: Ideal for patients whose primary disease burden is acute, life-threatening events requiring SGCs (6).
Patient-Centricity	Superior convenience identified in patient surveys; addresses psychological “treatment fatigue” (27, 28).	Adherence Optimization: Targeted at patients with a history of poor persistence or a psychological aversion to frequent injections (26, 27).
Healthcare Economics	Reduced clinic throughput requirements and administrative overhead (29).	Resource Allocation: Favored by health systems prioritizing streamlined outpatient care and high patient-to-nurse ratios (29, 30).
Comparative Limits	Mixed secondary outcomes (FEV1, QoL); absence of direct head-to-head non-inferiority data (8, 9, 25).	Symptom-Heavy Phenotype: Less suitable as first-line for patients prioritizing daily lung function gain or rapid symptomatic relief (19).

Synthesis of depemokimab’s clinical profile and its projected role within the Type 2 (T2) inflammatory treatment landscape. Strategic positioning is based on the integration of established anti-IL-5 efficacy (7, 13) with the logistical advantages of a twice-yearly (Q6M) dosing cycle (10). The table highlights that while depemokimab may not offer superior functional improvements (FEV1) over daily-focused biologics (16, 18), its value is maximized in exacerbation-prone populations where treatment adherence and systemic resource optimization are the defining priorities (27, 28).

Furthermore, in patients with an exacerbation-dominant phenotype, where the primary therapeutic goal is the avoidance of SGCs and their associated long-term complications, depemokimab offers a proven prophylactic strategy (6, 8). From a pharmacoeconomic and healthcare systems perspective, the shift toward a biannual administration model aligns with a broader movement toward streamlined outpatient care (28). By significantly reducing the administrative and logistical overhead associated with more frequent biologic administrations, depemokimab may allow healthcare systems to optimize resource utilization while maintaining high standards of disease suppression (28, 29).

Ultimately, depemokimab is positioned to impact the T2 biologic market not necessarily through a claim of superior efficacy over its anti-IL-5 peers, but by offering superior logistical feasibility and guaranteed adherence. Assuming that forthcoming head-to-head non-inferiority data confirm its effectiveness is comparable to existing standards of care (25), the Q6M regimen will likely cement its status as a valuable option. It provides a unique opportunity for patients to normalize their daily lives with the least frequent and most discreet management schedule available today, fundamentally changing the expectations for long-term maintenance in eosinophilic respiratory diseases.

## References

[1] Ambrosino P Marcuccio G Raffio G Formisano R Candia C Manzo F . Endotyping Chronic Respiratory Diseases: T2 Inflammation in the United Airways Model. Life (Basel). (2024) 14:899. doi: 10.3390/life14070899, PMID: 39063652 PMC11278432

[2] Fokkens WJ Lund VJ Hopkins C Hellings PW Kern R Reitsma S . European Position Paper on Rhinosinusitis and Nasal Polyps 2020. Rhinology. (2020) 58:1–464. doi: 10.4193/Rhin20.600, PMID: 32077450

[3] Brozek JL Bousquet J Baena-Cagnani CE Bonini S Canonica GW Casale TB . Global Allergy and Asthma European Network; Grading of Recommendations Assessment, Development and Evaluation Working Group. Allergic Rhinitis and its Impact on Asthma (ARIA) guidelines: 2010 revision. J Allergy Clin Immunol. (2010) 126:466–76. doi: 10.1016/j.jaci.2010.06.047, PMID: 20816182

[4] Chung KF Wenzel SE Brozek JL Bush A Castro M Sterk PJ . International ERS/ATS guidelines on definition, evaluation and treatment of severe asthma. Eur Respir J. (2014) 43:343–73. doi: 10.1183/09031936.00202013. Erratum in: Eur Respir J. 2014 Apr;43(4):1216. Dosage error in article text. Erratum in: Eur Respir J. 2018 Jul 27;52(1):1352020. doi: 10.1183/13993003.52020-2013. Erratum in: Eur Respir J. 2022 Jun 9;59(6):1362020. doi: 10.1183/13993003.62020-2013. PMID: 24337046

[5] Senna G Guerriero M Paggiaro PL Blasi F Caminati M Heffler E . SANI. SANI-Severe Asthma Network in Italy: a way forward to monitor severe asthma. . Clin Mol Allergy. (2017) 15:9. doi: 10.1186/s12948-017-0065-4, PMID: 28400707 PMC5385599

[6] Volmer T Effenberger T Trautner C Buhl R . Consequences of long-term oral corticosteroid therapy and its side-effects in severe asthma in adults: a focused review of the impact data in the literature. Eur Respir J. (2018) 52:1800703. doi: 10.1183/13993003.00703-2018, PMID: 30190274

[7] Akdis CA Arkwright PD Brüggen MC Busse W Gadina M Guttman-Yassky E . Type 2 immunity in the skin and lungs. Allergy. (2020) 75:1582–605. doi: 10.1111/all.14318, PMID: 32319104

[8] Jackson DJ Wechsler ME Jackson DJ Bernstein D Korn S Pfeffer PE . Twice-Yearly Depemokimab in Severe Asthma with an Eosinophilic Phenotype. N Engl J Med. (2024) 391:2337–49. doi: 10.1056/NEJMoa2406673, PMID: 39248309

[9] Gevaert P Desrosiers M Cornet M Mullol J De Corso E Keles Turel N . Efficacy and safety of twice per year depemokimab in chronic rhinosinusitis with nasal polyps (ANCHOR-1 and ANCHOR-2): phase 3, randomised, double-blind, parallel trials. Lancet. (2025) 405:911–26. doi: 10.1016/S0140-6736(25)00197-7, PMID: 40037388

[10] Dall'Acqua WF Kiener PA Wu H . Properties of human IgG1s engineered for enhanced binding to the neonatal Fc receptor (FcRn). J Biol Chem. (2006) 281:23514–24. doi: 10.1074/jbc.M604292200, PMID: 16793771

[11] Menzies-Gow AN Price DB . Clinical Remission in Severe Asthma: How to Move From Theory to Practice. Chest. (2023) 164:296–8. doi: 10.1016/j.chest.2023.03.001, PMID: 37558327

[12] Hellings PW De Corso E Backer V Bernal-Sprekelsen M Chan Y Conti DM . Remission in chronic rhinosinusitis with nasal polyps (CRSwNP). Rhinology. (2025) 63:239–41. doi: 10.4193/Rhin24.457, PMID: 39982222

[13] Nolasco S Crimi C . Depemokimab, the first ultra-long-acting anti-IL-5 monoclonal antibody for severe eosinophilic asthma. Med. (2024) 5:1452–5. doi: 10.1016/j.medj.2024.10.022, PMID: 39674169

[14] Singh D Fuhr R Bird NP Mole S Hardes K Man YL . A Phase 1 study of the long-acting anti-IL-5 monoclonal antibody GSK3511294 in patients with asthma. Br J Clin Pharmacol. (2022) 88:702–12. doi: 10.1111/bcp.15002, PMID: 34292606 PMC9290054

[15] Schalkwijk S Zecchin C Sen A Choi S Wang K Min J . Pharmacokinetics of Depemokimab Delivered by Safety Syringe Device or Autoinjector in Healthy Adults: A Phase 1, Single-Dose Study. Clin Pharmacol Drug Dev. (2025) 14:190–9. doi: 10.1002/cpdd.1506, PMID: 39876532 PMC11905874

[16] Kallieri M Papaioannou AI Loukides S . Mepolizumab for severe eosinophilic asthma. Expert Rev Respir Med. (2026) 20:13–25. doi: 10.1080/17476348.2025.2545571, PMID: 40782356

[17] Louis R Lommatzsch M Jackson DJ Menzies-Gow A Shavit A Cohen D . Advancing Remission in Severe Asthma With Benralizumab: Latest Findings, Current Perspectives and Future Direction. Clin Exp Allergy. (2025) 55:521–31. doi: 10.1111/cea.70083, PMID: 40444568 PMC12221862

[18] Bjermer L Lemiere C Maspero J Weiss S Zangrilli J Germinaro M . Reslizumab for Inadequately Controlled Asthma With Elevated Blood Eosinophil Levels: A Randomized Phase 3 Study. Chest. (2016) 150:789–98. doi: 10.1016/j.chest.2016.03.032, PMID: 27056586

[19] Pavord ID Bel EH Bourdin A Chan R Han JK Keene ON . From DREAM to REALITI-A and beyond: Mepolizumab for the treatment of eosinophil-driven diseases. Allergy. (2022) 77:778–97. doi: 10.1111/all.15056, PMID: 34402066 PMC9293125

[20] Bachert C Sousa AR Han JK Schlosser RJ Sowerby LJ Hopkins C . Mepolizumab for chronic rhinosinusitis with nasal polyps: Treatment efficacy by comorbidity and blood eosinophil count. J Allergy Clin Immunol. (2022) 149:1711–21. doi: 10.1016/j.jaci.2021.10.040, PMID: 35007624

[21] Castro M Corren J Pavord ID Maspero J Wenzel S Rabe KF . Dupilumab Efficacy and Safety in Moderate-to-Severe Uncontrolled Asthma. N Engl J Med. (2018) 378:2486–96. doi: 10.1056/NEJMoa1804092, PMID: 29782217

[22] Bachert C Mannent L Naclerio RM Mullol J Ferguson BJ Gevaert P . Effect of Subcutaneous Dupilumab on Nasal Polyp Burden in Patients With Chronic Sinusitis and Nasal Polyposis: A Randomized Clinical Trial. JAMA. (2016) 315:469–79. doi: 10.1001/jama.2015.19330, PMID: 26836729

[23] Menzies-Gow A Wechsler ME Brightling CE Korn S Corren J Israel E . DESTINATION study investigators. Long-term safety and efficacy of tezepelumab in people with severe, uncontrolled asthma (DESTINATION): a randomised, placebo-controlled extension study. Lancet Respir Med. (2023) 11:425–38. doi: 10.1016/S2213-2600(22)00492-1. Erratum in: Lancet Respir Med. 2023 Mar;11(3):e25. doi: 10.1016/S2213-2600(23)00048-6. PMID: 36702146

[24] Li W . Targeting the IL-4/IL-4R Axis in Th2 Inflammatory Diseases: A Review of Clinical Efficacy and Safety. J Inflammation Res. (2025) 18:17857–77. doi: 10.2147/JIR.S558065, PMID: 41458354 PMC12739947

[25] Chupp G Nagase H Skowasch D Devouassoux G Côté A Jackson DJ . NIMBLE Study Investigators. Switching to twice-yearly depemokimab from mepolizumab/benralizumab in severe asthma: A multicenter, randomized, double-blind, Phase 3A Clinical Trial (NIMBLE). Am J Respir Crit Care Med. (2026) 11:aamag031. doi: 10.1093/ajrccm/aamag031, PMID: 41738176 PMC13160935

[26] Yang M Chao J Fillbrunn M Mallya UG Wang MJ Franke L . Patient Preferences for Attributes of Biologic Treatments in Moderate to Severe Asthma: A Discrete Choice Experiment Study. Patient Prefer Adherence. (2022) :16:2649–2661. doi: 10.2147/PPA.S365117, PMID: 36176349 PMC9514297

[27] Maddux JT Inselman JW Jeffery MM Lam RW Shah ND Rank MA . Adherence to Asthma Biologics: Implications for Patient Selection, Step Therapy, and Outcomes. Chest. (2021) 159:924–32. doi: 10.1016/j.chest.2020.10.050, PMID: 33558205 PMC7965652

[28] Rose SJ Nakamura A Hossain MM Guilbert TW Ramsey RR . Real-World Adherence to Biologic Therapy for Severe Asthma in Children. J Allergy Clin Immunol Pract. (2025) 13:3316–23. doi: 10.1016/j.jaip.2025.09.020, PMID: 40998258 PMC12848759

[29] Available online at: https://www.ohe.org/publications/understanding-the-full-value-of-long-acting-therapies-is-less-more/.

[30] Murphy CL Awan S Sullivan MO Chavrimootoo S Bannon C Martin L . Major cost savings associated with biologic dose reduction in patients with inflammatory arthritis. Ir Med J. (2015) 108:19–21. 25702349

[31] Efficacy and Safety of Depemokimab Compared With Mepolizumab in Adults With Relapsing or Refractory Eosinophilic Granulomatosis With Polyangiitis (EGPA) (OCEAN) . Available online at: https://clinicaltrials.gov/study/NCT05263934.

[32] Depemokimab in Participants With Hypereosinophilic Syndrome, Efficacy, and Safety Trial (DESTINY) . Available online at: https://clinicaltrials.gov/study/NCT05334368.

